# Macular Pigment and Its Contribution to Vision

**DOI:** 10.3390/nu5061962

**Published:** 2013-05-29

**Authors:** Ekaterina Loskutova, John Nolan, Alan Howard, Stephen Beatty

**Affiliations:** 1 Macular Pigment Research Group, Department of Chemical and Life Sciences, Waterford Institute of Technology, Waterford, Ireland; E-Mails: jmnolan@wit.ie (J.N.); sbeatty@wit.ie (S.B.); 2 Institute of Vision Research, Whitfield Clinic, Waterford, Ireland; 3 Howard Foundation, Downing College, Cambridge University, Cambridge, CB22 5LA, UK; E-Mail: alan.howard@howard-foundation.com

**Keywords:** lutein, zeaxanthin, meso-zeaxanthin, visual performance, macular pigment

## Abstract

Three dietary carotenoids, lutein (L), zeaxanthin (Z) and meso-zeaxanthin (MZ) accumulate at the central retina (macula), where they are collectively referred to as macular pigment (MP). MP’s pre-receptoral absorption of blue light and consequential attenuation of the effects of chromatic aberration and light scatter are important for optimal visual function. Furthermore, antioxidant activity of MP’s constituent carotenoids and the same blue light-filtering properties underlie the rationale for its putative protective role for age-related macular degeneration (AMD). Supplementation with L, Z and MZ augments MP and enhances visual performance in diseased and non-diseased eyes, and may reduce risk of AMD development and/or progression.

## 1. Introduction

Vision is a process by which images of the external world can be interpreted by the seeing part of the brain. The retina facilitates transformation of light into an electrical signal and is an integral part of the visual system. 

The posterior pole of the retina is known as the macula. The central part of the macula, the fovea, is only about 2 mm in diameter, but has the highest concentration of light-sensitive cone photoreceptor cells, and is responsible for detailed central and color vision. The macula houses three carotenoid pigments, lutein (L), zeaxanthin (Z) and meso-zeaxanthin (MZ), which are collectively referred to as macular pigment (MP). 

In humans, L and Z are entirely of dietary origin, and are found in eggs and many types of brightly colored fruits and vegetables typical of a western diet [1]. The presence of MZ in foodstuffs is currently under investigation, but MZ is known to be produced in the macula following isomerization of retinal L [2].

Of the 42 dietary carotenoids, 14 are absorbed and used by the human body, yet only L, Z and MZ are found at the macula [3]. The preferential accumulation of L, Z and MZ at the site of sharpest vision is thought to be the result of an uptake mechanism that has evolved in response to the functional needs of the tissue (macula). MP optimizes visual performance in non-diseased eyes because of its pre-receptoral absorption of blue light and consequential attenuation of chromatic aberration and the adverse impact of light scatter (“veiling luminance”). As a result, and because of the known inter-individual variability of MP levels, optimal visual performance in non-diseased eyes is dependent upon optimal MP levels and a peaked spatial profile of this pigment. Furthermore, MP may also protect against age-related macular degeneration (AMD) because of the same blue light-filtering properties and because of the anti-inflammatory and antioxidant activities of MP’s constituent carotenoids [4,5]. 

## 2. Measures of Visual Function

Vision is a complex process, which includes resolving power, discernment of objects against a contrasting background (contrast sensitivity, or CS), depth perception, color discrimination, movement recognition, amongst other facilities. No single test reflects all of these parameters of visual function, but there are a number of techniques to assess different aspects of visual performance. The most widely used means of testing vision is known as visual acuity (VA), which measures spatial resolving power of the visual system at a 100% contrast. “Normal” VA is deemed to be the ability to discriminate symbols that are 1 arc minute apart. VA is primarily a function of the cones, a type of retinal photoreceptor cell that is also responsible for color vision, and which reaches peak density at the fovea (the central macula). It is important to note that the resolving power of the eye does not describe the ability to discern the foreground from the background within the field of view.

CS is a measure of the visual system’s ability to distinguish objects of dissimilar luminance, and is measured, and varies substantially, for different target sizes. CS relates directly to how well a person can perform tasks of everyday life, such as identifying faces, driving or reading [6]. CS declines with increasing age, and is a more sensitive indicator of visual dysfunction attributable to retinal disease than is VA [7]. In other words, measures of CS are more reflective of overall visual performance than is VA, in non-diseased and in diseased eyes.

## 3. The Process of Vision

The process of vision involves a metabolic response to a physical stimulus. Before the brain can construct an image, light entering the eye needs to be “bent”, or refracted, by the optical system in order to be focused on the retina, where it will be transformed into nerve signals, and transmitted to the visual processing center in the occipital cortex for ultimate perception of the stimulus.

### 3.1. The Optical System of the Eye

The optical system comprises the cornea, the iris and the lens, and is responsible for filtration, refraction and regulation of light intensity. First, light is incident upon the cornea, which filters out ultraviolet (UV) light, and this structure accounts for most of the eye’s refractive power. The amount of light passing through the lens is regulated by the pupil. Visible light is then further refracted by the lens, allowing a clear and sharp image to be formed on the retina in an emmetropic eye (emmetropia is the state of vision where an object at infinity is in sharp focus when the crystalline lens is in a neutral or relaxed state).

### 3.2. Optical Limitations: Glare, Chromatic Aberration, and Light Scatter

Several optical limitations of the visual system can adversely impact upon the quality of vision in the normal eye. It is likely that the ability to accumulate MP at the macula has evolved in order to attenuate the impact of some of these optical imperfections on image quality [8]. 

#### 3.2.1. Chromatic Aberration

Visible light is spectrally composed of differing wavelengths, from short (blue) to long (red). These wavelengths are refracted by the optical medias to different degrees, with the blue light being refracted substantially more and, therefore, being defocused at the retina. This phenomenon is known as chromatic aberration (CA), and is visually perceived as a bluish blur at the edge of a viewed object, and can substantially and adversely affect the quality of vision, and is reflected in reduced CS [9]. 

#### 3.2.2. Light Scatter

Particles suspended in the atmosphere and structures within the eye scatter light incident upon them. The lens and cornea cause 70% and 30% of light scattered by the eye, respectively, with the aqueous and vitreous contributions to light scatter being minimal. Importantly, blue light is scattered more than other wavelengths, and the resulting scattered blue light superimposes a bluish “veil” over the retinal image, referred to as veiling luminance [10]. Veiling luminance reduces image contrast and visibility, thereby impairing vision [11]. 

#### 3.2.3. Glare

Glare is yet another factor which can impair visual performance. There are two types of glare: discomfort glare and disability glare [11]. Discomfort glare is caused by distracting and/or uncomfortable intense light sources, and results in an instinctive desire to squint or look away from the light source. Disability glare, on the other hand, is caused by light scatter and consequential veiling luminance, and impairs visual performance, but is unassociated with discomfort. 

In summary, visible blue light is deleterious to the quality of the optical image formed at the retina, because shorter wavelengths are scattered more than other wavelengths, thereby resulting in glare disability and veiling luminance, and also because these short blue wavelengths are more defocused at the retina than other wavelengths, thereby causing chromatic aberration. Further, there are no blue-sensitive cones in the foveola, and blue visible light is therefore solely deleterious to high frequency spatial vision.

### 3.3. Visual Benefits of MP

Optically, MP is a blue light filter, with maximum absorption circa 460 nm, and screens out deleterious short-wavelength light. It has been shown that MPOD is positively related to the heterochromatic contrast thresholds (obtained by presenting the target with contrast grating stimulus on a blue, 460 nm, background), likely because MP’s preferential absorption of blue surround increases target detectability [12]. Under natural conditions, objects are often presented on short-wavelength background, such as blue sky and green leaves, meaning that the filtering properties of MP may be important for real-life vision. 

MP’s pre-receptoral filtration of blue light is believed to reduce the adverse impact of glare disability, light scatter and chromatic aberration, thereby optimizing CS [13,14,15]. It follows, therefore, that augmentation of MP would result in enhanced CS and improved glare disability. Indeed, as shown by clinical trials, this can be achieved by supplementation with a formulation containing the macular carotenoids [16,17,18]. Of interest, in a study by Loughman *et al.*, best visual outcomes were seen when the formulation contained all three of MP’s constituent carotenoids (MZ, L and Z in a (mg) ratio of 10:10:2) [18].

However, the visual benefits of MP are not restricted to the effects of its optical properties, reflected in a growing body of evidence that the macular carotenoids may have a favorable effect on neuronal processing [19,20]. These carotenoids have been shown to improve communication through cell-to-cell channels, modulate the dynamic instability of microtubules (structural units of neurons), and prevent degradation of synaptic vesicle proteins [21,22,23]. 

Rubin *et al.* have investigated the effects of supplementation with carotenoids (L, Z, lycopene and β-carotene) on plasma levels of these compounds, incidence of prematurity complications, levels of a biomarker of inflammation (C-reactive protein) and electroretinography (ERG) outcomes in preterm infants compared to human milk-fed term infants [24]. Supplementation with carotenoids increased plasma concentrations of these compounds into the range observed for term infants. Moreover, infants who received supplementation showed a significantly greater sensitivity response in rod photoreceptors than infants in the control group, suggesting that carotenoids have an initiative effect on rod function and, thereby, positively affect retinal development in infancy. As discussed by the authors, the proposed mechanism probably involves the same light-absorbing, antioxidant and anti-inflammatory activities of the macular carotenoids that have already been alluded to. Interestingly, levels of the biomarker of inflammation were lower following supplementation, and approximated those of the term infants. This finding is consistent with other reports on the anti-inflammatory properties of the macular carotenoids [4,5]. 

Indeed, Hammond *et al.* have shown that MP is positively related to a dynamic measure of visual performance, termed critical fusion frequency (CFF) thresholds, which is believed to also reflect post-receptoral processes [19]. In a further study by Renzi *et al.*, these findings were confirmed and expanded upon by measuring the more complete temporal contrast sensitivity function (TCSF) [20]. In this latter study, MPOD was found to be positively related to CFF thresholds and to TCSF, suggesting that MP may be important for central visual processing.

### 3.4. Neurophysiology of Vision

The process of phototransduction in the retina is essential for vision. Two types of photoreceptors, known as rods and cones, share responsibility for conversion of light into a neural signal. Phototransduction takes place in photoreceptor outer segment membranes, which are organized in stacks. Photopigments within these membranes consist of a light-absorbing chromophore, retinal, and a protein moiety, opsin. When activated by incident light, retinal undergoes photoisomerisation and converts from the 11-*cis* to the all-*trans* form, thus initiating a signal transduction cascade [25].

The photoreceptors are located in the outer layers of the neurosensory retina, which means that they are facing away from incident light. The explanation for this inverted design is that photoreceptors need to be in close contact with the retinal pigment epithelium (RPE), which plays an essential role in sustaining the visual phototransduction cycle. 

The RPE supplies photoreceptors with nutrients, and constantly restores the chromophore from the all-*trans* to 11-*cis* configuration, thereby ensuring regeneration of the visual pigment [26]. RPE also recycles shed photoreceptor outer segments, and other metabolic waste. In fact, the metabolism and resultant photo-oxidative damage in the photoreceptor cells is so high that the outer segments must completely renew themselves every 10 days [27].

#### Disease of the Retina

With the highest metabolic activity in the mammalian world and associated high oxygen consumption, the retina, a tissue rich in readily oxidizable polyunsaturated fatty acids (PUFAs), is an ideal environment for the production of, and damage by, reactive oxygen intermediates (ROIs) [28]. Exposure to light, especially high energy short-wavelength light, and the presence of photosensitizers (chromophores), further increase production of ROIs in this tissue [29,30]. Oxidative stress resulting from excessive production of ROIs, and consequential inflammation, are important in the pathogenesis of AMD [31]. 

### 3.5. Protective Role of MP for AMD

MP’s pre-receptoral filtration of blue light at the macula (where photoreceptors reach their peak concentration) is believed to protect the vulnerable central retina from oxidative injury by limiting light-induced generation of ROIs [32]. MP’s constituent carotenoids also contribute to the antioxidant defense system through their capacity to quench singlet oxygen and scavenge free radicals [33]. Moreover, these compounds may also attenuate the deleterious effects of chronic inflammation in the macular region [4,5]. 

Accordingly, it is biologically plausible that MP protects against AMD, and supplementation with the macular carotenoids could represent a strategy of preventing and/or delaying the onset of AMD or retarding progression of this disease [34]. 

## 4. Conclusion

There is firm evidence that MP is necessary for optimal visual function. Indeed, supplementation with MP’s constituent carotenoids can enhance visual performance in non-diseased and diseased eyes, with best results following supplementation with all three of MP’s constituent carotenoids (MZ, L and Z in a (mg) ratio of 10:10:2). Finally, there is a biologically plausible rationale whereby MP’s optical and antioxidant properties may reduce risk of AMD development and/or progression (as recently shown by AREDS2) [35].
